# Urologische Ausbildungsstationen – sinnvolle Antwort auf den Fachkräftemangel?

**DOI:** 10.1007/s00120-026-02853-0

**Published:** 2026-06-09

**Authors:** Christian P. Meyer, Rainer Treichel, Patrick Büttner, Yvonne Schmidgen, Stephan Degener, Jost Hohage, Simone Hyun, Friedrich-Carl von Rundstedt

**Affiliations:** 1 Universitätsklinik für Urologie, Ruhr-Universität Bochum, Klinikum Herford, Herford, Deutschland; 2 Universitätsklinik für Urologie, Universität Witten-Herdecke, Helios Klinikum Wuppertal, Wuppertal, Deutschland

**Keywords:** Nachwuchsgewinnung, Praktisches Jahr, Interprofessionelles Lernen, Medizinische Ausbildung, Patientensicherheit, Recruitment, Clinical clerkship, Interprofessional learning, Medical education, Patient safety

## Abstract

**Hintergrund/Ziele:**

Der akute Fachkräftemangel in der Urologie erhöht den Druck auf Lehre und Nachwuchsgewinnung. Interdisziplinäre Ausbildungsstationen (IAS) könnten ein Modell sein, um Studierende und Ärzt:innen klinisch besser auszubilden, Kompetenzen interdisziplinär zu fördern und dadurch indirekt dem Mangel entgegenzuwirken. Diese Übersichtsarbeit untersucht Konzept, Evidenz, Struktur und die spezifische Anwendung im urologischen Bereich.

**Material und Methoden:**

Systematische Literaturrecherche über PubMed, Google Scholar und bildungsspezifische Datenbanken (z. B. MedEdPublish, BMC Medical Education) bis Mitte 2025. Eingeschlossen wurden Arbeiten zu interdisziplinären/interprofessionellen Ausbildungsstationen, klinischen Lehreinheiten und Lehrkliniken, mit Fokus auf operative Fächer bzw. Urologie, aber auch Belege aus anderen Disziplinen.

**Ergebnisse:**

Historisch entwickelten sich Ausbildungsstationen aus Lehrkliniken und interprofessionellen Modellen des 20. Jahrhunderts. Der Aufbau beinhaltet gemeinsame Teams, rotierende Teilnehmer:innen, Verantwortung für Patientenversorgung unter Supervision sowie Curricula mit Lernzielen und Feedback-Strukturen. Wissenschaftliche Daten zeigen positive Effekte für klinische Kompetenz, Teamarbeit, Patientenzufriedenheit und möglicherweise eine Verringerung von Fehlern und Kosten. Spezifische Daten zur Urologie sind jedoch selten. In der Urologie existieren der Unterricht am Krankenbett (UAK), Famulaturen und klinische Wahlfächer, die Komponenten einer IAS aufweisen, aber selten ein durchgängig interdisziplinäres Stationsmodell.

**Schlussfolgerung:**

Die IAS bieten großes Potenzial zur Verbesserung der Ausbildung und könnten helfen, dem Fachkräftemangel entgegenzuwirken. Für die Urologie sind weitere empirische Studien erforderlich, darunter kontrollierte Vergleiche, Kosten-Nutzen-Analysen und Implementierungsstudien.

## Abstract (en)

**Background/Objectives:**

The pressing shortage of professionals in urology increases pressure on medical education and recruitment. Interdisciplinary teaching wards (ITWs) may offer a model to enhance clinical training, promote interdisciplinary competencies, and thereby help mitigate scarcity. This review explores the concept, evidence, structure, and specific application in urology.

**Materials and methods:**

Systematic literature search of PubMed, Google Scholar, and educational databases up to mid-2025. Inclusion criteria: reports on interdisciplinary/interprofessional teaching wards, clinical teaching units, clerkships, especially in surgical fields and urology; data on structure, outcomes, implementation.

**Results:**

Historically, teaching wards arose from clinical teaching hospitals and interprofessional education in the 20th century. Their structure typically includes multidisciplinary teams, rotating learners, supervised patient care, and curriculum with outcomes and feedback. Evidence shows benefits in clinical skills, teamwork, patient satisfaction, and a possible reduction of errors and costs. However, urology-specific data are sparse. In urology, clerkships, electives and subinternships incorporate certain aspects, but fully developed ITWs in urology are rare.

**Conclusion:**

ITWs hold significant promise for improving education and may contribute to alleviating the workforce shortage in urology. More empirical studies focused on urology are required, especially randomized controlled trials, economic analyses, and implementation studies.

## Hinführung

Der demographische Wandel und die zunehmende Spezialisierung im Gesundheitssystem führen zu Engpässen speziell in operativen Fächern wie der Urologie.

Zur besseren Quantifizierung des Fachkräftemangels zeigen aktuelle Analysen der Kassenärztlichen Bundesvereinigung (KBV) sowie Weiterbildungsumfragen einen zunehmenden Mangel an Ärzt:innen in Weiterbildung in operativen Fächern, insbesondere in der Urologie. Prognosen gehen davon aus, dass ein signifikanter Anteil der aktuell tätigen Urolog:innen in den kommenden Jahren altersbedingt ausscheiden wird [1–3]. Dieses trifft auf die zunehmende Alterung der Gesellschaft, die zu einer vermehrten Nachfrage nach medizinischen Leistungen führt. In der jetzigen Form ist das System nicht nachhaltig und muss neu gedacht werden. Die Voraussetzungen hierfür sind gegeben: Eine Mehrheit der Studierenden wünscht sich eine neu-definierte Rolle des Arztberufes in interprofessionellen Teams mit Delegation bisher ärztlicher Tätigkeiten [4].

Diese Übersichtsarbeit soll die Hypothese überprüfen, ob Ausbildungsstationen, bei denen Studierende und Ärzt:innen in Weiterbildung in interdisziplinären Teams klinische Verantwortung unter Supervision übernehmen, helfen, Kompetenzen früh zu fördern, Lehre mit Praxis zu verbinden, Nachwuchs nachhaltig zu binden und zu einer adäquaten Antwort auf den Fachkräftemangel beitragen können.

Die Ausbildung in der Medizin begann traditionell in Lehrkrankenhäusern und Lehrpraxen, die durch klare Hierarchien und eine fachliche Trennung geprägt waren. Im Laufe des 20. Jahrhunderts entwickelte sich das Konzept der Teaching Hospitals, in denen Lehre, Forschung und klinische Versorgung eng miteinander verzahnt sind. Parallel dazu entstanden interprofessionelle Ausbildungsprogramme [5, 6], bei denen verschiedene Berufsgruppen wie Pflegekräfte und Medizinstudierende gemeinsam ausgebildet wurden. Diese Ansätze waren eine Antwort auf die zunehmende Komplexität der Patientenversorgung. Lehrstationen oder „ward teaching units“ [7] wurden in Ländern wie Großbritannien und Australien etabliert, um Studierenden die aktive Mitarbeit in der Patientenversorgung zu ermöglichen. Die Verantwortung unter Supervision förderte frühzeitig klinische Kompetenz, sowohl in Diagnostik als auch in Therapie.

Im deutschsprachigen Raum haben Ausbildungsstationen ihre Wurzeln in klassischen Strukturen wie dem Praktischen Jahr (PJ) und dem Bedside Teaching, das bereits im 19. Jahrhundert etabliert wurde. Problemorientiertes Lernen (POL) und strukturierte klinische Ausbildungsmodule wurden insbesondere seit den 1990er-Jahren in den Curricula verankert. Die Weltgesundheitsorganisation (WHO [6]) betont in mehreren Positionspapieren die Bedeutung interprofessioneller Ausbildung als Schlüsselfaktor für die Verbesserung der Patientensicherheit und den Abbau von Versorgungslücken weltweit.

## Methodik

Für diese Übersichtsarbeit wurde eine systematische Literaturrecherche nach den Prinzipien der PRISMA-Leitlinien durchgeführt [8]. Die Suche erfolgte in PubMed, Google Scholar sowie in bildungsspezifischen Datenbanken (MedEdPublish, BMC Medical Education) bis Mitte 2025. Verwendet wurden die Suchbegriffe „interprofessional education“, „teaching ward“, „clinical training“, „urology“ und „workforce shortage“.

Eingeschlossen wurden Publikationen, die sich mit Ausbildungsstationen, interdisziplinären Lehrformaten oder strukturierten urologischen Ausbildungsprogrammen befassten. Zusätzlich wurden relevante Dokumente von Fachgesellschaften (z. B. DGU, BÄK, WHO, OECD) sowie Ausbildungsinformationen von Universitäten berücksichtigt.

**Abb. 1:**
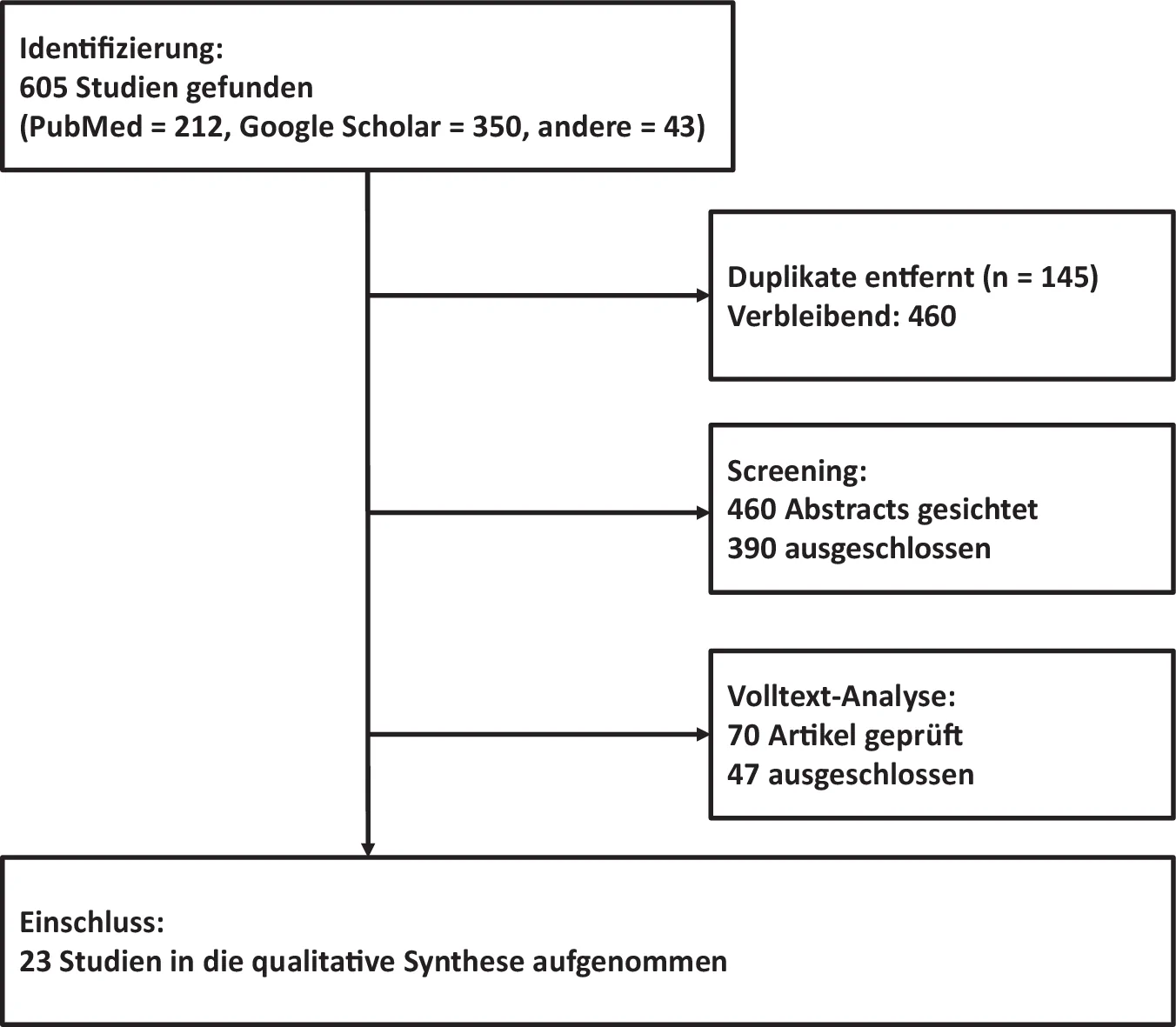
PRISMA-flow-Diagramm der Literaturrecherche. Darstellung der identifizierten, ausgeschlossenen und eingeschlossenen Studien nach systematischer Recherche

Insgesamt wurden 605 Artikel identifiziert. Nach Entfernung von Duplikaten (*n* = 145) verblieben 460 Titel und Abstracts, die gescreent wurden. Davon wurden 390 als nicht relevant ausgeschlossen. 70 Arbeiten wurden im Volltext analysiert, wovon 53 nicht die Einschlusskriterien erfüllten. Letztlich wurden 23 Arbeiten in die qualitative Synthese aufgenommen. Der Ablauf der Recherche ist im PRISMA-flow-Diagramm dargestellt (Abb. 1).

## Aufbau und Struktur von Ausbildungsstationen

Interdisziplinäre Ausbildungsstationen sind durch eine enge Teamstruktur geprägt, in die Ärzt:innen, Pflegekräfte, Therapeut:innen sowie Studierende verschiedener Fachrichtungen eingebunden sind [9]. Die Lernenden übernehmen abgestuft Verantwortung für Patienten unter Supervision erfahrener Fachärzt:innen bzw. Praxisanleiter:innen. Klare Curricula mit definierten Lernzielen, strukturierte Feedbackprozesse und regelmäßige Evaluationen sichern die Qualität der Ausbildung. Organisatorisch erfordert dies eine Station mit klarer Leitung, ausreichenden Ressourcen und einer engen Abstimmung mit den klinischen Abläufen. Durch kontinuierliche Evaluation und Qualitätssicherung können Effizienz und Lernerfolg überprüft und verbessert werden [5, 7, 10]. Ein zentrales Element des Aufbaus sind standardisierte Lernzielkataloge, die sich an den Nationalen Kompetenzbasierten Lernzielkatalogen Medizin (NKLM) orientieren. Prüfungsformate wie „objective structured clinical examinations“ (OSCE, oder „mini-clinical evaluation exercises“ (Mini-CEX, werden eingesetzt, um Lernfortschritte strukturiert zu bewerten. Interprofessionelle Zusammenarbeit umfasst die aktive Integration von Pflegekräften, Physiotherapeut:innen sowie Sozialdiensten in die Ausbildungsstruktur. Organisatorische Hürden ergeben sich häufig durch begrenzte finanzielle Ressourcen, fehlende Freistellung von Lehrpersonal und unklare rechtliche Rahmenbedingungen hinsichtlich der Verantwortung von Studierenden und Auszubildenden in der direkten Patientenversorgung.

Internationale Best-practice-Beispiele verdeutlichen den möglichen Aufbau von Ausbildungsstationen. Am Karolinska Institutet [11–17] in Schweden etwa übernehmen Studierende im Rahmen von „interprofessional training wards“ gemeinsam mit Pflegeschüler:innen die Betreuung von Patient:innen [12]. Die Mayo Clinic [14] in den USA hat ein Modell etabliert, das neben Medizin und Pflege auch Physiotherapie und Sozialarbeit aktiv einbindet. Qualitätssicherung erfolgt über regelmäßige Evaluationen und Patientenfeedback. In Deutschland wurden ab 2017 die ersten interprofessionellen Ausbildungsstationen, v. a. in der Allgemeinchirurgie, etabliert. Auch hier hat sich ein Konzept der kontinuierlichen Anleitung und Supervision klinischer Tätigkeiten durch den ärztlichen, pflegerischen Dienst bewährt. Der Einsatz erfolgt je nach Berufsgruppe und Ausbildungsstand in ein- bis 4‑wöchigen Blöcken [10].

Die positive Korrelation von klinischen Erfahrungen während des Studiums mit der späteren Fachwahl ist gut belegt [18–23]. Eine kontinuierliche und praxisnahe klinische Einbindung beeinflusst die Karriereentscheidung junger Ärzt:innen und den Verbleib in jeweiligen Fachrichtungen oder Versorgungsbereichen maßgeblich. Ergänzend unterstreichen Übersichtsarbeiten [22] sowie eine Cochrane-Analyse, dass interprofessionelle Ausbildungsformate die Berufszufriedenheit und Versorgungsqualität positiv beeinflussen [10, 22] (s. Tab. 1 und 2).

## Anwendung in der Urologie und Ausblick

Die urologische Ausbildung ist durch besondere Anforderungen geprägt [24]. Invasive Eingriffe wie Zystoskopien oder transurethrale Resektionen bieten ein hohes didaktisches Potenzial, setzen aber auch eine enge Supervision voraus. Die Breite der konservativen Therapien und das hohe Maß an Multiprofessionalität kennzeichnen zudem den hohen Anspruch an unser Fach.

Im deutschsprachigen Raum kommen die Studierenden außer über die regulären Lehrformate wie Unterricht am Krankenbett (UAK) oder Famulaturen nur (zu) selten mit der Urologie in Kontakt. Zwar enthalten diese einzelnen Elemente interdisziplinärer Ausbildungsstationen, bieten aber kein integratives Modell. Das Potenzial solcher Modelle liegt in der Möglichkeit, Studierende frühzeitig an operative Fertigkeiten heranzuführen, die interprofessionelle Teamarbeit zu fördern und die Bindung an das Fach zu stärken. Herausforderungen bestehen v. a. in der Ressourcenverfügbarkeit, der Sicherstellung der Patientenzufriedenheit und der Akzeptanz durch Klinikleitungen. Für die Zukunft sind empirische Studien notwendig, die den Nutzen, die Kosten und die langfristigen Effekte von Ausbildungsstationen in der Urologie systematisch untersuchen. Pilotprojekte könnten als wichtige Grundlage für eine breitere Implementierung dienen.

Aktuelle Daten der Deutschen Gesellschaft für Urologie e. V. (DGU [4]) zeigen, dass bis 2035 rund 40 % der derzeit tätigen Urolog:innen altersbedingt ausscheiden werden. Besonders betroffen sind ländliche Regionen, wo die Nachwuchsgewinnung ohnehin erschwert ist. Die OECD (Organisation for Economic Co-operation and Development) [22] prognostiziert, dass der Bedarf an Fachärzt:innen für urologische Erkrankungen weltweit bis 2040 um bis zu 60 % steigen könnte, getrieben durch den demografischen Wandel und die steigende Prävalenz urologischer Krankheiten wie Prostatakarzinom oder Urolithiasis. Politisch wird daher zunehmend gefordert, Ausbildungsstationen nicht nur als fakultative Lehrform, sondern als verpflichtenden Bestandteil des Medizinstudiums zu etablieren. Die Digitalisierung eröffnet neue Möglichkeiten: Künstliche Intelligenz unterstützt bereits heute bei Diagnostik und Bildauswertung, während Telemedizin und virtuelle Simulationen neue Lehrformate ermöglichen. Langfristig könnten hybride Modelle, die reale Ausbildungsstationen mit digitalen Lehrformen kombinieren, die Attraktivität und Effizienz der urologischen Ausbildung erheblich steigern (vgl. Tab. 2; Abb. 1).

**Tab. 1:** Wissenschaftliche Evidenz zu Ausbildungsstationen. Zusammenfassung ausgewählter Studien zu Effekten auf Patientensicherheit, Teamkommunikation, Berufszufriedenheit und Fachwahl.

Studie	Setting	Hauptergebnis
Hauer et al. (2008) [22]	USA, Medizinstudierende	Einflussfaktor auf Fachwahl (Innere Medizin)
Gelfand et al. (2002) [19]	USA, Chirurgie	Karriereentscheidungen beeinflusst
Beattie et al. (2024) [18]	UK, Longitudinal Clerkship	Einfluss auf spätere Tätigkeit
Goto et al. (2025) [21]	Japan, national	Verbesserte Kompetenzen, Fachbindung
Glynn et al. (2021) [20]	Irland, Alumni	Langfristiger Effekt auf Karrierepfade
Reeves et al. (2013) [7]	Cochrane Review	Bessere Teamkommunikation, Outcomes
Lancet Public Health (2020) [25]	Review	Verbesserte Patientensicherheit

**Tab. 2:** Outcome-orientierte Darstellung der Ausbildungsstationen. Zusammenfassung der berichteten Effekte auf Berufszufriedenheit, Fachwahlentscheidungen und Versorgungsqualität.

Outcome	Effekt	Quelle(n)
Berufszufriedenheit	Zunahme	[18], [19], [20], [21], [22], [23]
Fachwahlentscheidung	stärkere Bindung ans Fach	[18], [19], [20], [21], [22], [23]
Teamkommunikation	Verbesserung	[5], [7], [6]
Patientensicherheit	Verbesserung	[25], [7], [6]
Langfristige Bindung an Region/Fach	Nachweisbar	[18], [19], [22], [23]

Ein denkbares Modell für die Zukunft ist die „Urologische Lehrstation 2030“, die auf einer engen Verzahnung von Klinikalltag, digitalen Lehrmethoden und interprofessioneller Zusammenarbeit basiert. Simulationstraining, Virtual Reality und E‑Learning könnten ergänzend eingesetzt werden, um die Patientensicherheit zu erhöhen und Lernprozesse zu intensivieren. Langfristig könnte ein solches Modell nicht nur die Ausbildungsqualität verbessern, sondern auch die Attraktivität des Fachs Urologie steigern und damit aktiv dem Fachkräftemangel entgegenwirken. International existieren erste Modelle, in denen Urologie in interdisziplinäre Ausbildungsstationen eingebunden wurde, etwa in Kombination mit Nephrologie und Gynäkologie [26, 27]. Skandinavische Länder experimentieren mit Ausbildungsstationen, die Simulationsoperationen beinhalten, um invasive Techniken unter sicheren Bedingungen zu trainieren [28]. Diese Ansätze könnten auch für Deutschland wegweisend sein.

## Eigene Erfahrungen aus der Praxis

Als Antwort auf die genannten Herausforderungen wurden seit 2023 bzw. 2025 an den Standorten Wuppertal und Herford die ersten interprofessionellen urologischen Ausbildungsstationen etabliert. Die konzeptionelle Ausgestaltung erfolgte in enger Anlehnung an in der Literatur beschriebene Modelle interprofessioneller Ausbildungsformate.

Im Zentrum steht die interprofessionelle Zusammenarbeit zwischen Auszubildenden der Pflege und Medizinstudierenden. Ziel ist es, frühzeitig Interesse für das Fach Urologie zu wecken und die Bindung an das Fach zu stärken sowie potenzielle zukünftige Mitarbeitende für die eigenen Einrichtungen zu gewinnen.

Zur Gewährleistung einer praxisnahen Ausbildung bei gleichzeitiger Sicherstellung der Patientensicherheit wurde ein strukturiertes Supervisionskonzept implementiert. Dieses sieht die kontinuierliche Anwesenheit von Praxisanleitenden vor, die zugleich die Funktion der Stationsleitung übernehmen. Die Ausbildungsstation – als räumlich abgegrenzter Bereich einer regulären Station – ist montags bis freitags in Früh- und Spätschichten besetzt; außerhalb dieser Zeiten erfolgt die Versorgung durch die jeweilige Hauptstation.

Ein zentraler Bestandteil des Konzepts ist die engmaschige medizinische Supervision der PJ-Studierenden. Zur strukturierten Einbindung findet täglich eine Morgenvisite unter Leitung des zuständigen Stationsoberarztes sowie unter Beteiligung mindestens eines Arztes in Weiterbildung statt. Die PJ-Studierenden übernehmen hierbei wesentliche Anteile der Patientenversorgung, einschließlich der täglichen Stationsorganisation, der medizinischen Dokumentation sowie der Erstellung diagnostischer und therapeutischer Anordnungen, die nach ärztlicher Überprüfung freigegeben werden.

Ergänzend erfolgt einmal wöchentlich eine strukturierte, etwa einstündige Lehrvisite unter Leitung des Chefarztes oder eines Oberarztes. In diesem Rahmen werden ausgewählte Patient:innen vorgestellt, klinische Fragestellungen diskutiert und relevante Krankheitsbilder vertieft. Ziel ist es, Krankheitsbilder systematisch einzuordnen, prüfungsrelevantes Wissen für Medizinstudierende zu vermitteln und zugleich den Pflegeauszubildenden die zugrunde liegenden medizinischen Zusammenhänge verständlich darzustellen. Die Auswahl der Fälle erfolgt dabei so, dass zentrale Themenbereiche der operativen Urologie abgedeckt werden.

Zur weiteren Vertiefung der praktischen Ausbildung begleiten die PJ-Studierenden ihre Patient:innen auch in diagnostische und operative Eingriffe und assistieren bei entsprechenden Prozeduren. Zur Förderung des interprofessionellen Verständnisses erhalten auch Pflegeauszubildende Einblicke in endoskopische Verfahren sowie operative Abläufe im Operationssaal. Im Rahmen der Rotation sollen sie zudem grundlegende praktische Fertigkeiten, wie beispielsweise die Anlage eines transurethralen Blasenverweilkatheters, erlernen.

Die Ausbildungsstation hat sich seit ihrer Einführung in mehrfacher Hinsicht bewährt:

- Das PJ-Tertial gewinnt durch einen klar definierten eigenen Aufgabenbereich deutlich an Attraktivität.
- PJ-Studierende erhalten die Möglichkeit, sich frühzeitig klinisch zu profilieren und sich im Anschluss gezielt um eine Weiterbildungsstelle zu bewerben.
- Die Urologie gewinnt an Sichtbarkeit und Attraktivität gegenüber der Pflege, sowohl für stationäre Pflegekräfte als auch für potenzielle Mitarbeitende im Funktionsbereich.

Insgesamt stellt die Ausbildungsstation neben der Erweiterung didaktischer Möglichkeiten ein vielversprechendes Instrument der strukturierten Nachwuchsgewinnung dar. Sie ermöglicht eine praxisnahe Auseinandersetzung mit dem Fach und eröffnet frühzeitig berufliche Perspektiven. Der zentrale Erfolgsfaktor liegt dabei in einem Ausbildungskonzept, das interprofessionelles Arbeiten auf Augenhöhe fördert und konsequent berufsgruppenübergreifend umsetzt.

Ob diese Initiative langfristig zu einer nachhaltigen Nachwuchsgewinnung beiträgt, bleibt Gegenstand der aktuell begleitenden wissenschaftlichen Evaluationen.

## Fazit für die Praxis

- Ausbildungsstationen stellen ein wirkungsvolles Instrument dar, um klinische Fertigkeiten, Teamarbeit und interdisziplinäre Kompetenzen frühzeitig zu fördern.
- In der Urologie sind zwar bereits einzelne Lehrelemente etabliert, ein vollumfängliches interdisziplinäres Stationsmodell existiert jedoch bislang nicht.
- Um dem Fachkräftemangel wirksam zu begegnen, bedarf es zusätzlicher Projekte und Studien, die die Effekte solcher Stationen auf Ausbildung, Patientenversorgung und Nachwuchsgewinnung systematisch bewerten.
- Kliniken sollten prüfen, wie sie Ressourcen wie Betten, Personal und Supervision bereitstellen können, um die Umsetzung von Ausbildungsstationen zu ermöglichen. Dabei sind Qualitätssicherung, regelmäßiges Feedback und Evaluation ebenso wichtig wie die Schaffung von Anreizen für Lehrende und Institutionen.

## Data Availability

Alle dieser Arbeit zugrunde liegenden Daten sind in diesem Artikel enthalten.
